# dTGS: Method for Effective Components Identification from Traditional Chinese Medicine Formula and Mechanism Analysis

**DOI:** 10.1155/2013/840427

**Published:** 2013-12-18

**Authors:** Ji Luo, Yinglong Ren, Hao Gu, Yi Wu, Yun Wang

**Affiliations:** ^1^Beijing University of Chinese Medicine, School of Chinese Pharmacy No. 6, Zhonghuan South Road, Wangjing, Chaoyang, Beijing 100102, China; ^2^Pennsylvania State University Erie, The Behrend College, School of Engineering, 5101 Jordan Road, Erie, PA 16563, USA

## Abstract

Because of the complexity of the components in Traditional Chinese Medicine formula (TCM formula), it is still a challenge to identify its effective components, to elucidate the mechanism of the components, and to discover the relationship between components and therapy objectives. In this paper, a method called directed TCM grammar systems (dTGS) for effective component identification was proposed using entity grammar systems (EGS) as the theoretical framework. The component-disease relationship of a TCM formula (i.e., Bai-Hu decoction plus Wasting-Thirsting formula, BHDWT) and one disease (i.e., type 2 diabetes mellitus) treated with it was studied, and the effective component groups (ECGs) were identified. 19 compounds were found acting on 20 proteins in type 2 diabetes mellitus (T2D) disease network, and 15 compounds were determined as the candidate effective components. Results indicated that this method can be used to identify the effective components and provide an innovative way to elucidate the molecular mechanism of TCM formulas.

## 1. Introduction

The components in Traditional Chinese Medicine formula (TCM formula) were very complex and their molecular mechanism was unclear. For the treatment of one disease, some components may be favorable and others may not. Identification of favorable components and analysis of their action mechanisms will benefit the optimization of cultivation condition, processing technology, extraction process, and new drug development. At present, experiment screening was still the main method for the identification of effective components and effective component groups (ECGs). For example, high-performance liquid chromatography-mass spectrometry (HPLC-MS) was used to analyze the active constituents of Xiao-Xu-Ming decoction [1]; drosophila transgenic models were used to identify combinatorial drug, such as suberoylanilide hydroxamic acid (SAHA) and geldanamycin, for the treatment of Huntington's disease [2]; cell-based assays technology was used to screen two-component combinations for the treatment of cancer, infectious diseases, and CNS disorders [3]. However, the results identified through experimental screening were limited, due to the complexity of components and the high cost associated with experiments.

Recently, computational systems biology was used to study TCM because of the technical advantages of studying large and complex systems and the relative lower cost compared to experimental screening. The applications of complex network analysis techniques, in particular, led to many new findings. For instance, microarray technology and connectivity maps were integrated into the research of molecular mechanisms of Si-Wu decoction (composed of four herbs: Radix Rehmanniae preparata, Radix Angelicae Sinensis, Rhizoma Ligustici Chuanxiong, and Radix Paeoniae Alba) [4]. Multilayer map of “Phenotype network-Biological network-Herb network” was applied to uncover the underlying network systems of TCM syndromes and herb formulas [5]; the drug-target network was implemented to elucidate the mechanism of one TCM formula for the treatment of T2D [6]. Although those applications of computational systems biology in the study of TCM formulas are still in the exploratory stages, they demonstrate the feasibility of integrating the biological network and the experiences in traditional medicines for the analysis of TCM formulas.

To date, graph theory is a primary approach for network research [7]. It is viable to study a network graph composed of dozens or hundreds of nodes through visual inspection. However, it is not practical to analyze a network containing massive nodes or complex relations between them, even with the help of three-dimensional display techniques. Most methods developed for complex networks, such as the path-length method and the nodes-distribution method [8], focus on the topological structure instead of the specific relationship between nodes. Therefore, it is still challenging to study a complex disease network with intercrossing pathways and to understand the final effects of the components of TCM formulas based on the biological signal pathways. In addition, those biological effects may be ambiguous (positive through one pathway, while negative through the other one) plus some proteins affected by the components of TCM which have not been identified as disease targets. In order to solve these issues and to discover components with positive effect from TCM formulas, we proposed a new approach called directed TCM grammar systems (dTGS) to identify effective components from TCM formula based on an entity grammar system (EGS). In dTGS, the TCM component-protein network and the disease network are viewed as grammar systems and the ECGs can be identified through syntax rules. Bai-Hu decoction plus Wasting-Thirsting formula (BHDWT) was selected as an example in this paper to illustrate the basic idea of the method.

## 2. Materials and Methods

### 2.1. Definition of dTGS Model in the Framework of EGS

EGS is a formal grammar system that aims at complex biological system modeling [9]. Because of its scalable feature, EGS has already been used to establish the flow graph models of chemical processes [10] and to illustrate the mechanisms of TCM [11]. The details for establishing a specific EGS were described in [9] and are briefly summarized here.

An entity grammar system *G* is a quintuple, *G* = (*V*_*N*_, *V*_*T*_, *F*, *P*, *S*), whereas *V*_*N*_ ∪ *V*_*T*_ = *V*, *V*_*N*_ is a finite set of nonterminal symbols, *V*_*T*_ is a finite set of terminal symbols, and *V*_*N*_∩*V*_*T*_ = Φ, *F* is a finite set of relations for *V*; *P* is a set of rules to deduce relationships between entities, and *S* is the starting entity.

dTGS has the same structure as EGS: set *V* contains different types of nodes (compounds, proteins, T2D, apoptosis, inflammation, etc.); set *F* contains different types of relationships between adjacent nodes; set *P* defines the rules to derive the relationship of nodes, as described by the following:

(1) $$\begin{array}{c} V=V_{1}\cup V_{2}\cup V_{3}\cup V_{4}. \end{array}$$

*V*_1_ is the set of the compounds in TCM formulas, *V*_2_ is the set of proteins in the disease network on which the compounds in TCM formulas act directly, *V*_3_ is the set of the rest proteins in the disease network, and *V*_4_ is the set of nonprotein nodes in the disease network (e.g., T2D, apoptosis, inflammation, etc.). Consider

(2) $$\begin{array}{c} F=\left\{\text{cp}\left(X,Y,Z\right),\text{pp}\left(X,Y,Z\right),\text{draw}\left(X,Y,Z\right),\text{tag}\left(X\right)\right\}. \end{array}$$

In cp(*X*, *Y*, *Z*), *X* ∈ *V*_1_, *Y* ∈ *V*_2_, *Z* ∈ {pos, neg}; in pp(*X*, *Y*, *Z*), *X*,  *Y* ∈ *V*_3_ ∪ *V*_4_, *Z* ∈ {pos, neg}; in draw(*X*, *Y*, *Z*), *X*,  *Y* ∈ *V*, *Z* ∈ {pos, neg}; in tag(*X*), *X* ∈ *V*.

cp(*X*, *Y*, *Z*) defines that *X* (compound in TCM formulas) acts on *Y* (protein directly reacts to compound) with an effect described in *Z*. pp(*X*, *Y*, *Z*) defines that *X* (protein) acts on *Y* (protein) with an effect described in *Z*. tag(*X*) labels nodes of interest. draw(*X*, *Y*, *Z*) extracts tagged nodes, and their relationships defined in cp(*X*, *Y*, *Z*) or pp(*X*, *Y*, *Z*) in dTGS; *E*(*V*, *F*) refers to all entities composed by the elements in *V*, with the structure cp(*X*, *Y*, *Z*), pp(*X*, *Y*, *Z*), draw(*X*, *Y*, *Z*), or tag(*X*). Consider

(3) $$\begin{array}{c} P=P_{1}\cup P_{2}\cup P_{3}\cup P_{4}\cup P_{5}\cup P_{6},\\ P_{1}=\left\{\text{pp}\left(X,Y,\text{pos}\right),\text{pp}\left(Y,M,\text{pos}\right)\Rightarrow \text{pp}\left(X,M,\text{pos}\right)\right\},\\ P_{2}=\left\{\text{pp}\left(X,Y,\text{neg}\right),\text{pp}\left(Y,M,\text{neg}\right)\Rightarrow \text{pp}\left(X,M,\text{pos}\right)\right\},\\ P_{3}=\left\{\text{pp}\left(X,Y,\text{pos}\right),\text{pp}\left(Y,M,\text{neg}\right)\Rightarrow \;\text{pp}\left(X,M,\text{neg}\right)\right\},\\ P_{4}=\left\{\text{pp}\left(X,Y,\text{neg}\right),\text{pp}\left(Y,M,\text{pos}\right)\Rightarrow \;\text{pp}\left(X,M,\text{neg}\right)\right\},\\ P_{5}=\left\{\text{pp}\left(X,Y,Z\right),\text{tag}\left(X\right)\Rightarrow \text{draw}\left(X,Y,Z\right),\text{tag}\left(Y\right)\right\},\\ P_{6}=\left\{\text{cp}\left(X,Y,Z\right),\text{tag}\left(X\right)\Rightarrow \text{draw}\left(X,Y,Z\right),\text{tag}\left(Y\right)\right\}. \end{array}$$

*P*_1_ ∪ *P*_2_ ∪ *P*_3_ ∪ *P*_4_ is the set of rules to deduce the eventual effects of chemical compounds on the disease. *P*_1_ indicates that if the effect of *X* to *Y* is positive and the effect of *Y* to *M* is positive too, then the effect of *X* to *M* is positive. Similar derivations are defined in *P*_2_, *P*_3_, and *P*_4_. They may be used as many times as necessary to the final disease node. *P*_5_ and *P*_6_ are the rules to extract the nodes for the network. They can also be used to draw the subnetwork when the original networks are too complicated to analyze. *P*_5_ indicates that if *X* (protein) acts on *Y* (protein) with the effect of *Z* and *X* is tagged, then this relation is extracted and *Y* is also labeled for further derivation. *P*_6_ is similar to *P*_5_ except that the starting entity is chemical compound *X*. In the construction of network, *P*_6_ will be used once and *P*_5_ may be used as many times as necessary to the target.

**Figure 1:**
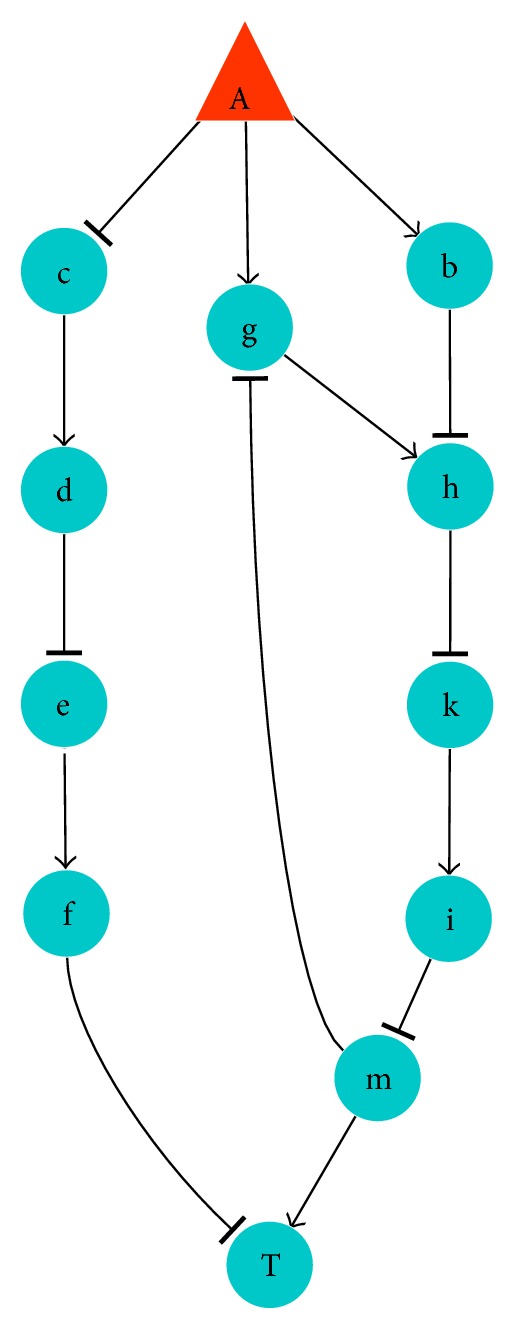
Network used in dTGS for deduction. Red triangle node: chemical compound; blue circle node: protein; “→”: one chemical component or one protein enables the expression of the next protein, raises the expression, or enhances the activity of the next protein. “⊣”: one chemical component or one protein inhibits the expression of the next protein, lowers the expression, or weakens the activity of the next protein.

The modes of action between nodes include the positive (pos) and the negative (neg) effects. If we define the positive effect as “1” and the negative effect as “−1,” the ultimate influence of intervention will depend on the product of each step in the whole signal pathway. For example, compound A in Figure 1 influences protein T through 3 paths. The effect of A to T is negative through the path “A-c-d-e-f-T” and the path “A-b-h-k-i-m-T.” This effect is uncertain through the third path “A-g-h-k-i-m-T,” taking into account the negative effect from the feedback path m to g. In this paper, we neglected the effects produced through feedback because the role of feedback is expected to regulate the magnitude of effect but not to alter the overall mode of action of effect. So, the effect is positive through the third path. After the effects of one compound on the ultimate node (i.e., T in Figure 1) through all signal pathways are determined, we can select the effective compounds based on the desired effect. If the desired effect on the ultimate node is positive, the compounds with positive effects through all pathways will be selected as active components. The compounds with negative effects through all pathways will be ruled out. The components with both effects through different pathways need further analysis on molecular mechanism. If their undesired effects can be countered by other compounds, they may also be selected as effective components to be used together with the countering compounds. The opposite analysis will be done if the desired effect on the ultimate node is negative. Consider

(4) $$\begin{array}{c} S=S_{1}\cup S_{2}. \end{array}$$

*S*_1_ is the set of entities with structure cp(*X*, *Y*, *Z*) or pp(*X*, *Y*, *Z*) in biological network of disease, which are the background for deduction. *S*_2_ is the set of labeled compounds or proteins, expressed by tag(*X*). *S*_2_ is the initial conditions for deduction.

### 2.2. Data for Construction of Component-Protein Network of BHDWT Formula

For decades, BHDWT has been used to treat T2D at the Beijing Guang-An-Men Hospital [12]. BHDWT has a positive effect on blood glucose control and symptom control for some patients in the early stages of T2D. The BHDWT formula consists of eight herbs, including gypsum, *Anemarrhena asphodeloides* Bunge, rehmannia dried rhizome, radix trichosanthis, *Ophiopogon japonicus* Ker Gawl, *Coptis chinensis* Franch, *Scutellaria baicalensis* Georgi, and *Glycyrrhiza uralensis*.

The components of the BHDWT formula came from the Traditional Chinese Medicines Database (TCMD) [13], the State Administration of Traditional Chinese Medicine Basic Information Database (http://dbshare.cintcm.com/ZhongYaoJiChu/), and *A Handbook on the Analysis of the Active Composition in Traditional Chinese Medicine* [14].

The compound-targeted proteins were derived from the STITCH system (http://stitch.embl.de/) [15]. By entering the names or identifiers of compounds or proteins of interest, STITCH provides the list of proteins with matching or higher confidence score that the user specified, up to the number the user specified. The required confidence score represents the possibility of interaction between the entities. In order to obtain more general results, the parameter of the required confidence score was set higher than 0 and the interacting entities number was set to be 500. The interacting entities with clear mode of action (positive or negative) were chosen for further analysis.

### 2.3. Data for the Construction of T2D Biological Network

To construct the T2D network, we used the data collected from the Kyoto Encyclopedia of Genes and Genomes (KEGG) and therapeutic targets database (TTD). KEGG lists the signal pathways related to T2D and TTD lists the chemical components used to treat T2D. The biological network of T2D (Figure 3) was constructed using the signal pathways from KEGG, the chemical components from TTD, and the positive or negative relationship between targets and T2D from STITCH. The networks were visualized with the software Cytoscape [16].

## 3. Results and Discussion

### 3.1. Compound-Protein Network of BHDWT Formula and the Biological Network of T2D

**Figure 2:**
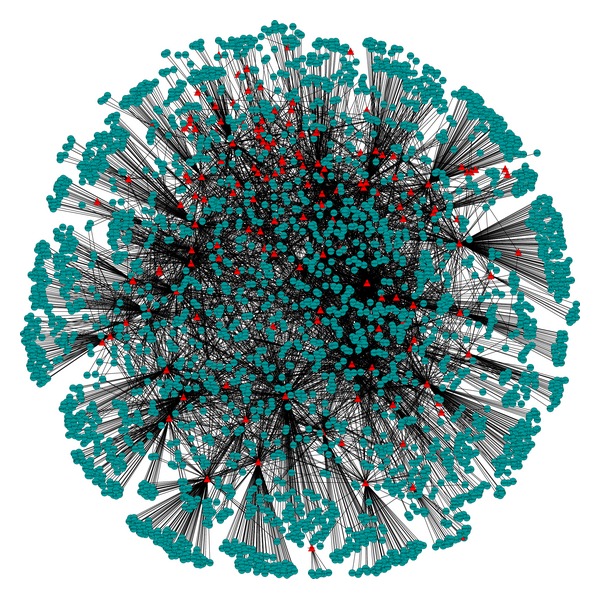
Compound-protein network of BHDWT. Red triangle node: compound in BHDWT; blue circle node: protein.

**Figure 3:**
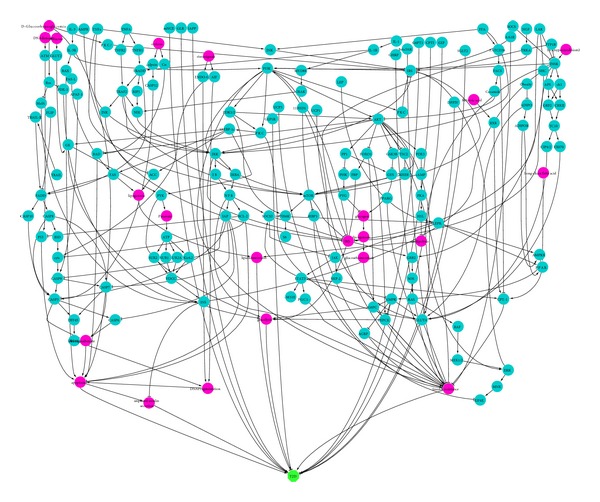
Biological network of T2D. Blue circle node: protein; purple circle node: nonprotein node; green octagon nodes: type 2 diabetes; “→”: one node enables the expression of the next node, raises the expression, or enhances the activity of the next node; “⊣”: one node inhibits the expression of the next node, lowers the expression, or weakens the activity of the next node.

We first constructed the component-protein network of BHDWT using the data from Section 2.2 (Figure 2, additional file 1 in Supplementary Material available online at http://dx.doi.org/10.1155/2013/840427). In this network, there are 144 compounds and 2865 proteins. The red triangle nodes represent the compound in BHDWT and the blue circle nodes represent the proteins. The biological network of T2D (Figure 3) was derived from the data in Section 2.3 using *P*_5_ and *P*_6_ defined in Section 2.1, with T2D as the ultimate node. Biological network of T2D contains 146 proteins. The effects of each compound in BHDWT on T2D were derived using *P*_1_, *P*_2_, *P*_3_, and *P*_4_ rules defined in Section 2.1.

### 3.2. The Effect of Chemical Components on T2D

**Figure 4:**
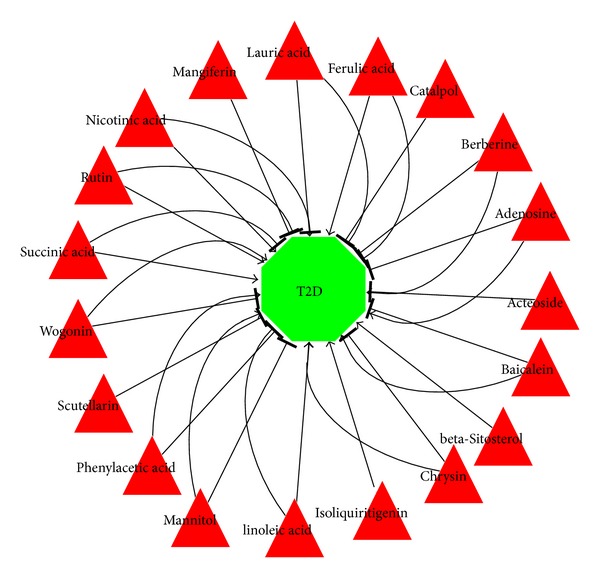
The effect of compound of BHDWT on T2D. Red triangle node: chemical compound; green octagon nodes: type 2 diabetes; “→”: one compound has the positive effect on T2D; “⊣”: one compound has the negative effect on T2D.

The ultimate effects of each compound of BHDWT on T2D can be found by combining Figures 2 and 3 through dTGS. We applied rule *P*_6_ for each compound node in Figure 3 as *X*, with the linked protein with *X* to be *Y* in *P*_6_ rule. As a result, all the compounds in Figure 2 that have clear modes of action on linked protein were labeled. The proteins presented in both Figures 2 and 3 were also labeled by applying *P*_6_. With the labeled protein as *X* and the connected node as *Y*, the pathway describing the relationship can be extracted by applying *P*_5_. Those compounds' ultimate effects on T2D (Figure 4) can be derived through *P*_1_, *P*_2_, *P*_3_, and *P*_4_. Totally, 45 compounds in 7 TCMs (except gypsum) showed effects on 61 proteins in the T2D biological network. Among 45 compounds, 19 (additional file 2) have a clear mode of action (positive or negative) recorded in STITCH. Three kinds of effect were found: positive, negative, and bidirectional effects. The desired effect on T2D is negative. Among these 19 compounds, *β*-sitosterol, isoliquiritigenin played a positive effect on T2D (i.e., negative effect on the treatment of T2D) and four compounds (i.e., scutellarin, catalpol, mangiferin, and acteoside) have negative effects on T2D (i.e., positive effect on the treatment of T2D). The rest of the 13 compounds show bidirectional effects and will be studied further in the next section.

### 3.3. Extraction of the Subnet and Effective Components

**Figure 5:**
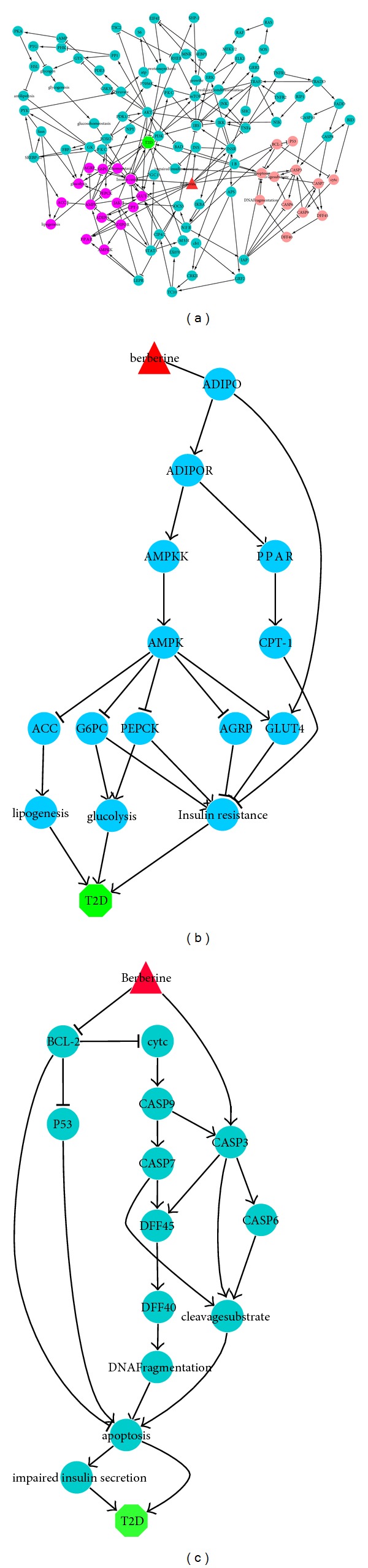
Sub-network affected by berberine. (a) Sub-network of berberine acting on six proteins. (b) Sub-network of berberine acting on ADIPO. (c) Sub-network of berberine acting on BCL-2 and caspase 3.

For each of the 13 bidirectional compounds, we extracted the subnetworks affected by each of them to study their effects in more detail. The method of extracting the subnetwork has been explained in Section 2.1. We found through sub-networks that the negative effect of rutin and phenylacetic acid on T2D originates from the feedback pathways, being not expected to override the positive effect through direct pathways. So, these two compounds were not considered as candidate effective components. Some other bidirectional components have a complex sub-network. For example, berberine acts on six proteins. The derived sub-network including all six proteins is too complex for further analysis (Figure 5). Therefore, we derived six sub-networks including one or two proteins reacting with berberine. Two of those sub-networks were shown in Figures 5(b) and 5(c). Through analyzing these six sub-networks, we found that berberine's negative effect on T2D arises from the direct pathway. Finally, all 13 bidirectional components except rutin and phenylacetic acid were selected as candidate effective components. They together with the other four negative compounds (i.e., scutellarin, catalpol, mangiferin, and acteoside) form 15 candidate effective components combinations and will be screened further in the next section.

### 3.4. Combination of Candidate Effective Components

**Figure 6:**
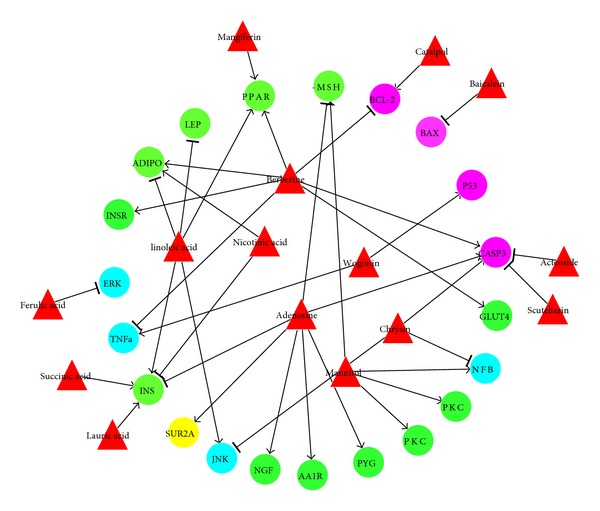
Relationship between effective components of BHDWT and reactive proteins. Red triangle node: active chemical component. Green nodes: proteins in the first category (related to insulin resistance). Purple nodes: proteins in the second category (related to apoptosis). Blue nodes: proteins in the third category (related to both insulin resistance and apoptosis). Yellow nodes: proteins in the fourth category (related to insulin secretion).

In this section, we try to find out which proteins each of the 15 effective components affects. This will help us figure out how to combine those components to achieve optimal results. According to the literature, insulin resistance and impaired insulin secretion are two major etiological factors of T2D [17], and *β*-cell apoptosis was considered as one reason for the impaired insulin secretion [18]. Therefore, we divided the proteins into four categories. The first category is only related to insulin resistance; the second category is only related to apoptosis; the third category is related to both insulin resistance and apoptosis; the fourth category is only related to insulin secretion. Then, the effects of each active component were screened according to the category of proteins (Figure 6). For instance, mangiferin acts on insulin resistance related proteins (PPARa) and catalpol acts on apoptosis related proteins (BCL-2); hence, the combination of mangiferin and catalpol was predicted to treat T2D by ameliorating insulin resistance and inhibiting apoptosis. It is worth noting that Figure 6 missed one protein in additional file 2, that is, CASP9. This is due to the fact that beta-sitosterol, the only compound which enables CASP9, was ignored because of beta-sitosterol's positive effect on T2D. The rest of the compounds in Figure 6 did not act on CASP9.

Some of the findings revealed in Figure 6 are consistent with numerous studies on the treatment of T2D. Ferulic acid showed antidiabetic effects in experiments on diabetic mice [19]. Mangiferin exhibited the potential to improve blood lipids in T2D [20]. Baicalein was demonstrated to protect pancreatic beta-cells from apoptosis and ameliorates hyperglycemia in a mouse model of T2D [21]. The experiment in vitro indicated that berberine can improve glucose consumption (GC) over 30% when the concentration is above 5 × 10^−6^ mol/L; at the same time, berberine also depressed cell growth remarkably at the same concentration [22]. This finding was consistent with our analysis that berberine promotes cell apoptosis by promoting caspase 3 and inhibiting BCL-2.

The compounds that have multiple and counterpart pathways in Figure 6 were still selected as candidate effective components to treat T2D because their unfavorable effects may not be dominant or counteracted by other compounds, as demonstrated in clinical practice. For example, some physicians have used berberine to treat hyperglycemia agent in China for many years [23].

Figure 6 also discloses some information useful for designing or analyzing component combination. Although the hepatotoxicity or pancreotoxicity (typically resulting from enhanced cell apoptosis) induced by berberine has never been observed in clinics, Figure 6 indicated that its toxicity may be counteracted while constructing drug combinations with the components that can inhibit cell apoptosis, such as catalpol, scutellarin, acteoside, and baicalein. In clinical practice, BHDWT was used to treat early stages of T2D when the main disease factor is insulin resistance [8]. This can be explained by several effective components acting on green nodes (i.e., proteins related to insulin resistance) in Figure 6. Similar effects to suppress the insulin resistance can also be achieved by the combinations of some of these effective components according to Figure 6, such as (i) the combinations of berberine and mangiferin, (ii) the combinations of berberine and catalpol (or scutellarin or acteoside or baicalein), and (iii) of berberine, mangiferin compination and catalpol (or scutellarin or acteoside or baicalein). Some of these combinations have been validated by the work from other researchers: the combination of berberine and mangiferin was granted a patent [24], and the combination of berberine and catalpol [25] has been filed for a patent. All of the results indicate that TCM formula plays its role through synergistic effects of multiple components.

## 4. Conclusions

This paper proposed dTGS as an innovative method to study TCM formulas. It integrates the research achievements from three fields: TCM chemistry, drug discovery, and the network biology. The findings include the action trends of chemical components against one disease (T2D) and the active component combinations from BHDWT formula. It can also be applied on other TCM formulas to benefit the research on the mechanism of TCM formulas.

In addition, our work would benefit the development of fixed-dose combinations. Nowadays, drug combinations or fixed-dose combinations (FDCs) are widely used in the treatment of complex diseases because of the low cost and the clinical efficiency. TCM formulas, due to their characteristics of multicomponents, multitargets, and multipath effects, may embody some component combinations or combination principles beneficial to the design of drug combination. Our method provides a systemic approach to reveal those principles.

Last but not least, our method provided a novel idea for network analysis. Our method is different from the primary approach in network research (e.g., graph theory) in that we proposed a series of inference rules derived from the relationship of the nodes and provided a new theoretical framework for analyzing the complex network. The feasibility of this theoretical framework was proved by its success to identify the effective component combinations in TCM formulas.

## Supplementary Material

Table S1*：* BHDWT network database
Tables S2*：*19 chemical components and proteins affected by them
