# Supplementary material for: dTGS: Method for Effective Components Identification from Traditional Chinese Medicine Formula and Mechanism Analysis
Source: Evid Based Complement Alternat Med. 2013 Dec 18;2013:840427. doi: 10.1155/2013/840427 (PMC3878852; doi:10.1155/2013/840427)
Supplement: Supplementary file 1 — Table S1： BHDWT network database Tables S2：19 chemical components and proteins affected by them [file 840427.f1.zip › description.docx]

Additional file 1：BHDWT network database

The data in additional file 1 was used to construct the component-protein network of BHDWT. The name of eight herbs included in the BHDWT formula were listed in the first column.The name of the compounds included in the eight herbs were listed in the second column. The name of proteins effected by the compounds were listed in the thired column.The proteins code obtained from the database of Uniprot were listed in the fourth column.

Additional file 2：19 chemical components and proteins affected by them

The data in additional file 2 was the initial data used to pursue. Those 19 components in the second column have a clear mode of action to the proteins in the third column components. The mode of action recorded in STITCH was listed in the fourth column involving two kinds of activation and inhibition. The data in the fifth column was the correlation score which represent the possibility of effect of component to the protein. In order to obtain more general results, we didn't limit the correlation score in this paper.
